# SARS-CoV-2 enterocolitis with persisting to excrete the virus for approximately two weeks after recovering from diarrhea: A case report

**DOI:** 10.1017/ice.2020.87

**Published:** 2020-03-19

**Authors:** Tomohiro Hosoda, Mitsuo Sakamoto, Hideaki Shimizu, Nobuhiko Okabe

**Affiliations:** 1 Department of Infectious Disease, Kawasaki Municipal Kawasaki Hospital; 2 Kawasaki City Institute for Public Health

*To the Editor*—In December 2019, a novel corona virus (SARS-CoV-2) was first isolated from patients in Wuhan, China. Since then, the outbreak has rapidly evolved mainly in Wuhan and Hubei Province and outside China.^1^ The outbreak on a large cruise ship docked in Yokohama, south of Tokyo, in January 2020 was the largest outbreak outside China. Person-to-person transmission of the virus has led to an epidemic of coronavirus disease 2019 (COVID-19). Throat swabs are used for screening or diagnostic purposes to identify individuals infected with this virus or possible carriers. Reverse transcriptase–polymerase chain reaction (RT-PCR) tests may have lower sensitivity when throat swabs are used than with nasal swab samples.^2^

An 81-year-old Japanese woman presented with a ~6-day history of abdominal pain, watery diarrhea, and a mild sore throat. However, she denied any fever and respiratory symptoms. She was transferred to our hospital from the aforementioned cruise ship (the site of the largest outbreak of SARS-CoV-2 in Japan) for evaluation and treatment of her abdominal symptoms. Her past medical history was unremarkable other than total gastrectomy for gastric cancer 5 years prior to presentation. Abdominal computed tomography on admission revealed acute enterocolitis without ileus or pneumonia. RT-PCR tests performed on throat swabs obtained at the time of transfer from the cruise and on day 4 of hospitalization showed negative results for SARS-CoV-2. However, RT-PCR testing of a stool sample obtained on day 2 of hospitalization showed positive results (2,000 copies per well), and she was diagnosed with acute enterocolitis secondary to COVID-19. Healthcare workers at our hospital performed contact and droplet precautions as essential components of patient care. She recovered from the diarrhea on day 4of hospitalization; however, RT-PCR testing of the stool sample continued to be positive on day 15 of hospitalization (200 copies per well). Negative results of the stool samples were obtained on days 16 and 17 of hospitalization.

Some patients in Wuhan, China, presented with diarrhea prior to the onset of fever and dyspnea.^3^ SARS-CoV-2 may be isolated from a diarrheal stool sample, which could cause person-to-person transmission.^4^ In our patient, the lack of gastric acid (attributable to the patient’s history of total gastrectomy) could have resulted in the detection of SARS-CoV-2 in her stool sample. Clinicians should be mindful of the fact that COVID-19 may manifest as enterocolitis in patients without respiratory tract infection, and they should provide thorough instruction in hand hygiene because patients, even after recovering from enterocolitis due to COVID-19, could continue to excrete the virus for weeks.
